# Neuroprotection or Increased Brain Damage Mediated by Temperature in Stroke Is Time Dependent

**DOI:** 10.1371/journal.pone.0030700

**Published:** 2012-02-17

**Authors:** Miguel Blanco, Francisco Campos, Manuel Rodríguez-Yáñez, Susana Arias, José Fernández-Ferro, José Carlos Gómez-Sánchez, José Castillo

**Affiliations:** 1 Department of Neurology, Clinical Neurosciences Research Laboratory, Hospital Clínico Universitario, University of Santiago de Compostela, Santiago de Compostela, Spain; 2 Department of Neurology, Hospital Clínico Universitario, Salamanca, Spain; University of South Florida, United States of America

## Abstract

The control of temperature during the acute phase of stroke may be a new therapeutic target that can be applied in all stroke patients, however therapeutic window or timecourse of the temperature effect is not well established. Our aim is to study the association between changes in body temperature in the first 72 hours and outcome in patients with ischemic (IS) and hemorrhagic (ICH) stroke. We prospectively studied 2931 consecutive patients (2468 with IS and 463 with ICH). Temperature was obtained at admission, and at 24, 48 and 72 hours after admission. Temperature was categorized as low (<36°C), normal (36–37°C) and high (>37°C). As the main variable, we studied functional outcome at 3 months determined by modified Rankin Scale.

Temperature in stroke patients is higher than in controls, and increases gradually in the first 72 hours after stroke. A positive correlation between temperature and stroke severity determined by NIHSS was found at 24 and 48 hours, but not at admission or 72 hours. In a logistic regression model, high temperature was associated with poor outcome at 24 hours (OR 2.05, 95% CI 1.59–2.64, p<0.0001) and 48 hours (OR 1.93, 95% CI 1.08–2.34, p = 0.007), but not at admission or 72 hours.

Temperature increases in patients with stroke in the first 72 hours, with the harmful effect of high temperature occurring in the first 48 hours. The neuroprotective effect of low temperature occurs within the first 24 hours from stroke onset.

## Introduction

Demographic changes and improvement in quality of health care systems in Western countries are conditioning an increase in the incidence and prevalence of ischemic and hemorrhagic cerebrovascular diseases. These serious consequences, together with vascular dementia, are expected to increase in the next decades, with no further options in prevention and treatment expected in the near future.

The control of vascular risk factors, especially hypertension, and probably the use of statins remain the most effective preventive treatments. Pharmacological (intravenous or intra-arterial) or mechanical reperfusion therapy are the most effective treatments during the acute phase of ischemic stroke (IS) and are associated with good outcome in 50–70% of cases [1]. However, it is difficult to apply these treatments in more than 10% of patients due to the short therapeutic window. There is currently no effective treatment for intracerebral hemorrhage (ICH).

The control of body temperature is becoming one the most promising neuroprotective treatment strategies for acute IS and ICH. In fact, in animal models of ischemia, reduction of body temperature has been observed to act as a one of the best neuroprotective strategies observed to date [2], [3].

In agreement with the clear experimental evidence, it was observed that the control of body temperature during the acute phase of IS and ICH is a therapeutic opportunity associated with good outcome in 10–25% of cases [4], [5], [6], [7], [8], [9], [10], [11]. However, to date, there is no clear clinical data in adequately large sample sizes which determine the effect of temperature, in relation to time, on outcome after ischemic stroke or hemorrhage [12], or the best moment to induce the control of temperature with neuroprotective results.

With this objective, in the present clinical study, we have analyzed and studied the association between changes in body temperature within the first 72 hours after stroke and the evolution of brain damage by looking at the inflammatory response and outcome at 3 months in a large series of patients with IS and ICH. In addition, we have tried to determine the time window for starting the control of body temperature.

## Methods

### Studied population

Since October 2004, we prospectively included all patients with IS and non-traumatic ICH admitted to the Neurology Department of the *Hospital Clínico Universitario de Santiago de Compostela* in the BICHUS registry, approved by the Research Ethics Committee of Galicia (CEIC). In November 2010, CEIC approved the analysis of this registry to study the influence of temperature in the first 72 hours in stroke outcome, and its possible association with systemic inflammation. Written informed consent was obtained from each patient or from their relatives after full explanation of the procedures.

From December 2004 to December 2010, 3873 patients were included for the analysis. Patients that had transient ischemic attack (TIA) (312 patients), transferred to other hospitals during the acute phase (17 patients), died by causes other than vascular (19 patients), lacked basal temperature registration (209 patients), lacked inflammatory markers (61 patients) and/or lacked follow-up at 3 months (375 patients) were excluded. Finally, 2931 patients (2468 with IS and 463 with ICH) were deemed valid for the analysis.

Patients were treated according to the Stroke Unit protocol of the *Hospital Clínico Universitario de Santiago de Compostela*. All patients with axillary temperature >37.5°C were treated with metamizol (2 g intravenous) or paracetamol (500 mg orally) every 6 hours (although treatment with metamizol and paracetamol were used to control fever, the condition of hypothermia was not induced in any of the patients recruited). All non-anticoagulated patients with IS received 100 mg/day of aspirin or 75 mg/day of clopidogrel if contraindication to aspirin was present. When reperfusion therapy was administered, antithrombotic treatment was delayed 24 hours.

In order to obtain a control group we recorded axillary temperature from 282 relatives of neurological outpatients, matched by sex and age.

### Studied variables

The following basal variables were included in this study: age, sex, type of stroke, previous history of vascular risk factors, blood pressure and glucose levels at admission and time from stroke onset.

In accordance with previous clinical studies [11] and following the Stroke Unit protocol of our hospital, after drying the armpit, the axillary temperatures were measured by professional nurses, after a 5 minute waiting time using mercury in glass thermometers. Temperature was obtained at admission and every 6 hours during the first 72 hours, which means that temperature was measured at 2 a.m., 8 a.m., 2 p.m. and 8 p.m. for 72 hours in every patient. Basal temperature and the highest temperature reading during the first 24, 48 and 72 hours were considered for the analysis. Temperature was analyzed as a continuous variable and categorized into three groups: low (<36°C), normal (36–37°C) and high temperature (>37°C).

Leukocyte count, fibrinogen, high-sensitive C-reactive protein (hs-CRP) and erythrocyte sedimentation rate (ESR) were analyzed as inflammatory variables. Systemic inflammation was considered present when at least two of the following results were observed: leukocyte count >10.000/mmc, fibrinogen >500 mg/dL, hs-CRP >3 mg/L and ESR >20 mm/h.

All patients underwent cranial computed tomography (CT) on admission. In patients with IS, control CT was obtained at 48–72 hours. In patients treated with rtPA, control CT was obtained 24 hours after starting treatment. Lesion volume was measured [13] in control CT in patients with IS and in CT on admission in patients with ICH.

Stroke subtype was classified according to TOAST criteria [14] in IS and as hypertensive, amyloid, in relation with antiplatelet/anticoagulant treatment, secondary to arteriovenous malformation or others in ICH.

The use of reperfusion therapy, the inclusion in clinical trials and the presence of infections during the first 72 hours were considered for the analysis. Certificated neurologists evaluated neurological deficit at admission, 24, 48 and 72 hours and at 3 months using NIHSS. Functional outcome was evaluated using modified Rankin Scale (mRS) at 3 months (±15 days) considering mRS <3 as good outcome and mRS ≥3 as poor outcome.

### Statistical analysis

Results are expressed as percentages for categorical variables and as mean ± SD or median [quartiles] for continuous variables, depending on whether or not the distribution was normal. Percentages were compared using the chi-square test. To compare continuous variables, Student t test for normally distributed variables or Mann-Whitney test for variables without normal distribution was conducted. ANOVA was used for comparison among several quantitative variables. Pearson's or Spearman coefficients determined the bivariate correlation. Several models of logistic regression analysis determined the influence of temperature on outcome at 3 months, after adjusting for those variables that were statistically significant (p≤0.05) in univariate analysis. The results were expressed as adjusted odds ratios with a confidence interval of 95%. Statistical analysis was conducted using SPSS 16.0 for Macintosh.

## Results

No differences in age (71.6±13.9 vs. 72.4±12.6 years, p = 0.316) or sex (male sex 62.8 vs. 58.1%, p = 0.145) were found between control group and patients.

**Figure 1:**
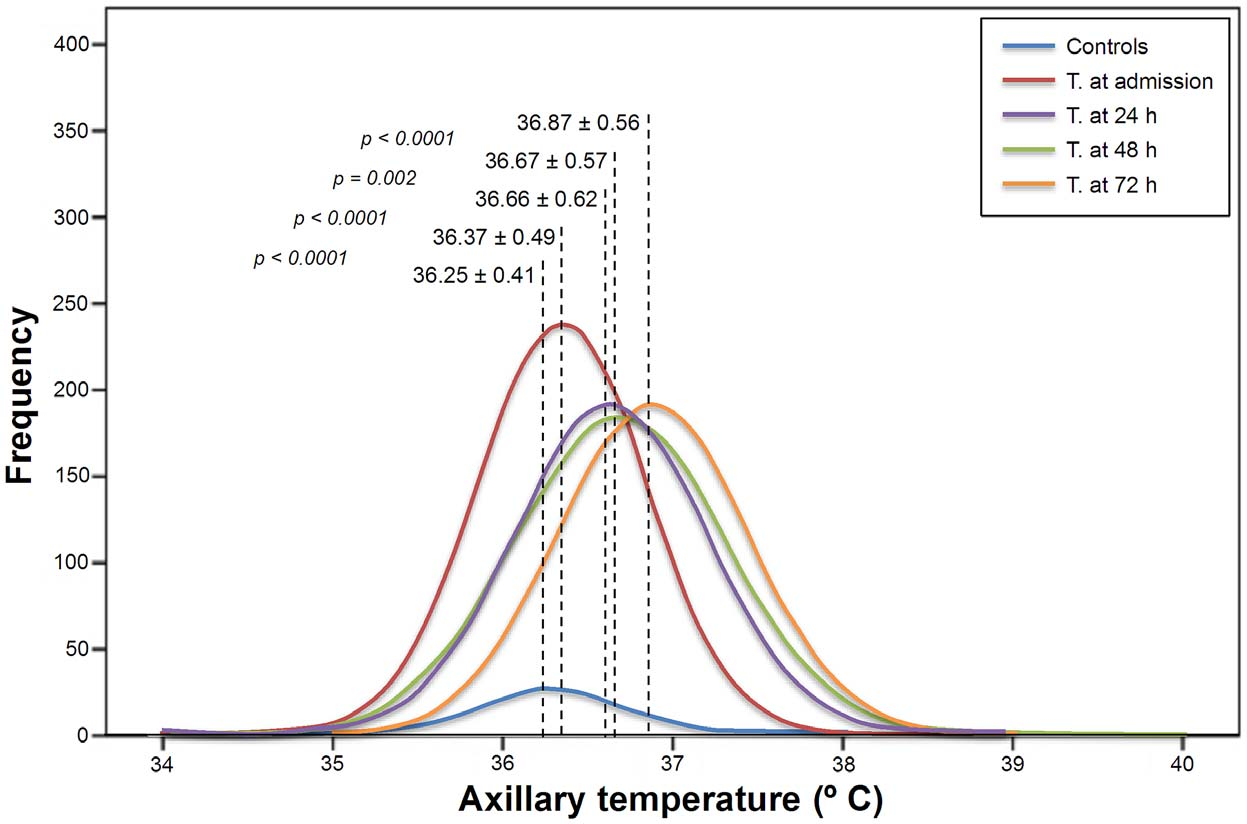
Normal distribution of temperatures in the control group and patients on admission and the maximum temperature in the first 24 hours, between 24 and 48 hours, and between 48 and 72 hours.

Temperature in control group was 36.25±0.41°C. Temperatures in stroke patients were 36.37±0.49°C on admission, 36.66±0.62°C at 24 hours, 36.67±0.57°C at 48 hours and 36.87±0.56°C at 72 hours. All temperatures were significantly higher than the previous one (p<0.0001 on admission in relation with control group, p<0.0001 at 24 hours in relation to admission, p = 0.002 at 48 hours compared to 24 hours and p<0.0001 at 72 hours in relation to 48 hours), as shown in (**Figure 1**). High temperature was present in 9.4% of patients on admission and this percentage increased over time (22.9% at 24 hours, 24.8% at 48 hours and 33.3% at 72 hours).

**Figure 2:**
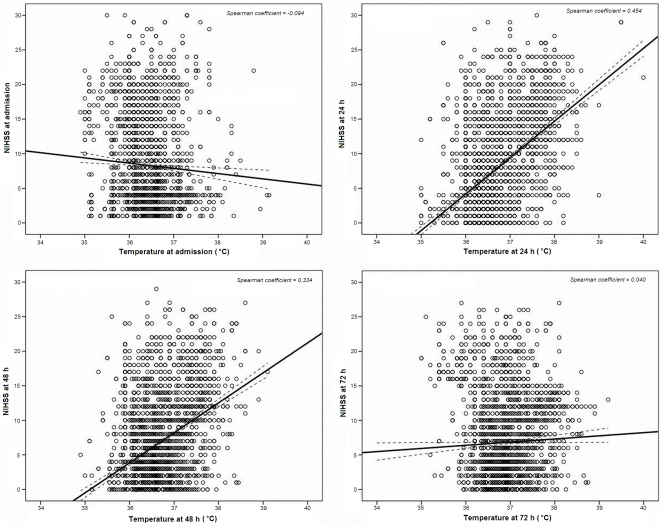
Scatterplot and regression lines with CI 95% between NIHSS and axillary temperature at admission, 24, 48 and 72 hours. The association between temperature and the severity of stroke is significant at 24 and 48 hours, but not on admission or at 72 hours.

Correlation between axillary temperature and stroke severity determined by the NIHSS was not demonstrated on admission (Spearman coefficient = −0.094, p = 0.064), although a significant correlation exists at 24 hours (Spearman coefficient = 0.454, p<0.0001) and at 48 hours (Spearman coefficient = 0.334, p<0.0001). This association disappears at 72 hours (Spearman coefficient = 0.040, p = 0.103) (**Figure 2**).

Poor outcome at 3 months was observed in 1118 patients (38.14%) and 361 patients died (12.31% of total). Patients with poor outcome had significantly higher temperatures at every time period analyzed (p<0.0001). The univariate analysis for outcome at 3 months is shown in **Table 1**.

**Table 1:** Univariated analysis: outcome at 3 moths (good outcome = modified Rankin Scale <3; poor outcome = modified Rankin Scale ≥3).

	Good outcome n = 1813	Poor outcome n = 1118	p
Age, years	70.7±12.8	75.2±11.7	<0.0001
Male, %	63.5	49.3	<0.0001
History of hypertension, %	39.5	45.2	0.003
History of diabetes, %	17.3	16.2	0.447
History of hyperlipidemia, %	22.2	21.1	0.490
Alcohol consumption, %	7.8	7.8	1.000
Tobacco consumption, %	11.4	8.1	0.004
Prior peripheral arterial disease, %	3.3	3.0	0.828
Prior coronary heart disease, %	7.6	8.8	0.265
Prior atrial fibrillation, %	10.3	16.8	<0.0001
Prior heart failure, %	1.3	3.1	0.001
Prior carotid disease, %	0.3	0.1	0.417
Prior TIA, %	0.9	2.2	0.006
Prior antiplatelet treatment, %	15.6	17.5	0.165
Prior anticoagulant therapy, %	3.8	17.5	0.013
Latency time, hours (n = 2325)	8.5±10.1	7.4±7.8	<0.0001
Axillary temperature on admission, °C (n = 2931)	36.3±0.5	36.5±0.4	<0.0001
Axillary temperature at 24 h, °C (n = 2895)	36.4±0.5	36.9±0.7	<0.0001
Axillary temperature at 48 h, °C (n = 2842)	36.5±0.4	36.9±0.7	<0.0001
Axillary temperature at 72 h, °C (n = 2672)	36.8±0.4	36.9±0.7	<0.0001
Axillary temperature on admission categorized			<0.0001
-Low, % (n = 474)	19.4	11.0	
-Normal, % (n = 2160)	71.1	77.8	
-High, % (n = 297)	9.5	11.2	
Axillary temperature at 24 hours categorized			<0.0001
-Low, % (n = 564)	24.6	10.9	
-Normal, % (n = 1666)	67.0	41.8	
-High, % (n = 664)	8.4	47.3	
Axillary temperature at 48 hours categorized			<0.0001
-Low, % (n = 196)	5.6	9.2	
-Normal, % (n = 1941)	81.1	45.9	
-High, % (n = 704)	13.4	44.9	
Axillary temperature at 72 hours categorized			<0.0001
-Low, % (n = 133)	2.0	10.2	
-Normal, % (n = 1648)	71.2	45.1	
-High, % (n = 890)	26.8	44.8	
Antipyretic treatmient during 48 hours, %	3.5	25.6	<0.0001
Infection within 48 h, %	2.5	15.7	<0.0001
NIHSS on admission (n = 2931)	4 [2], [6]	14 [7], [18]	<0.0001
NIHSS at 24 h (n = 2895)	3 [1], [6]	14 [9], [18]	<0.0001
NIHSS at 48 h (n = 2843)	2 [0, 5]	13 [10], [17]	<0.0001
NIHSS at 72 h (n = 2701)	2 [0, 5]	13 [9], [16]	0.115
Difference NIHSS 24 h−NIHSS 0 h	−1 [−2, 0]	0 [0, 1]	<0.0001
Difference NIHSS 48 h−NIHSS 24 h	0 [−2, 0]	0[−1,0]	<0.0001
Difference NIHSS 72 h−NIHSS 48 h	0 [0, 0]	0 [0, 0]	0.115
SBP on admission, mm Hg (n = 2931)	152.6±27.6	154.1±27.9	0.005
DBP on admission, mm Hg (n = 2931)	82.7±15.0	82.4±16.6	0.487
Glucose on admission, mg/dL (n = 2816)	139.3±62.9	146.9±63.4	0.001
Inflammation, %	13.9	51.3	<0.0001
-Leukocyte count, ×10^3^/mL (n = 2912)	8.1±2.6	9.8±3.1	<0.0001
-Fibrinogen, mg/dL (n = 2815)	433.6±95.1	507.6±123.9	<0.0001
-High-sensitive C-reactive, mg/L (n = 2611)	2.2±9.2	6.9±22.9	<0.0001
-Erythrocyte sedimentation rate, mm/h (2636)	19.9±18.6	34.4±25.8	<0.0001
Type of stroke			0.029
-Ischemic stroke, % (n = 2468)	85.4	82.3	
-Intracerebral hemorrhage, % (n = 463)	14.6	17.7	
TOAST			<0.0001
-Aterotrombotic, %	27.1	25.4	
-Cardioembolic, %	25.9	37.6	
-Lacunar, %	16.9	5.9	
-Undetermined, %	28.4	27.5	
-Other, %	1.7	3.4	
Non-lacunar vs lacunar stroke			<0.0001
-Non-lacunar, %	83.1	94.1	
-Lacunar, %	16.9	5.9	
Intracerebral hemorrhage			0.715
-Hypertensive, %	49.1	46.2	
-Amyloid, %	10.9	13.2	
-Antiplatelet/anticoagulant treatment, %	17.0	20.8	
-Arteriovenous malformation, %	2.3	2.0	
-Other, %	20.8	17.8	
Infart volume at 48–72 h, mL (n = 1407)	15.8±31.3	62.9±67.6	<0.0001
morrhage volume on admission, mL (n = 435)	23.7±37.2	59.2±62.7	<0.0001
Thrombolytic therapy, %	3.8	6.7	0.001
Inclusion in clinical trial, %	5.8	9.6	<0.0001

In logistic regression models, the effect of temperature was determined as a continuous variable in the four periods of time in relation to outcome at 3 months, adjusted for baseline variables significant in univariate analysis (age, sex, hypertension, smoking, atrial fibrillation, heart failure, previous TIA, anticoagulation, time from symptom onset to admission, systolic blood pressure, glucose levels, inflammation, NIHSS and temperature in every time period analyzed). The analysis showed no association with the basal body temperature (OR 1.10, 95% CI 0.98–5.13, p = 0.087), and with temperature at 72 hours (OR 0.98, 95% CI 0.76–1.26, p = 0.886), but an association was found at 24 hours (OR 2.05, 95% CI 1.59–2.64, p<0.0001) and 48 hours (OR 1.93, 95% CI 1.08–2.34, p = 0.007).

Two separate logistic regression models were performed for temperature at 24 and at 48 hours, and outcome. For patients with IS (lacunar vs. non-lacunar), variables included were infarct volume, use of thrombolytic therapy and inclusion in a clinical trial. For patients with ICH, variables included were volume on admission and inclusion in a clinical trial. A significant correlation between temperature at 24 hours and outcome at 3 months was found for patients with IS (OR 1.73, 95% CI 1.19–2.52, p = 0.004) and ICH (OR 2.16, 95% CI 1.14–4.01, p = 0.018). At 48 hours, a significant correlation still exists for patients with IS (OR 2.29, 95% CI 1.62–3.30, p = 0.001) but not for those with an ICH (OR 1.47, 95% CI 0.23–3.94, p = 0.133).

**Figure 3:**
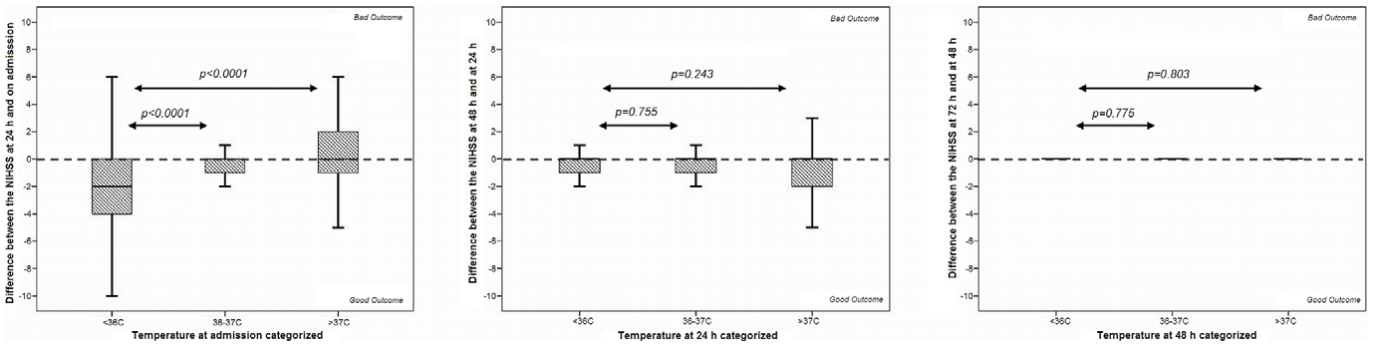
Differences in NIHSS score every 24 hours in patients with low, normal and high temperatures. In patients with low temperature (<36°C) within 24 hours a significant decrease in the NIHSS is observed.

To analyze the possible neuroprotective effect of low temperature, we determined the relationship between modifications of the NIHSS score in every period compared to the previous ones. The changes were only significant in the first 24 hours (NIHSS 24 hours - NIHSS on admission in the group of patients with low basal temperature was −2 [−4, 0], in patients with normal temperature 0 [−1, 0] and in patients with high temperature 0 [−1, 2], p<0.0001, **Figure 3**). In the group of patients with low temperature, temperature at admission was significantly associated with good outcome at 3 months (OR 0.84, 95% CI 0.50 to 0.94, p<0.0001), but this association was not demonstrated at 24, 48 or 72 hours.

**Figure 4:**
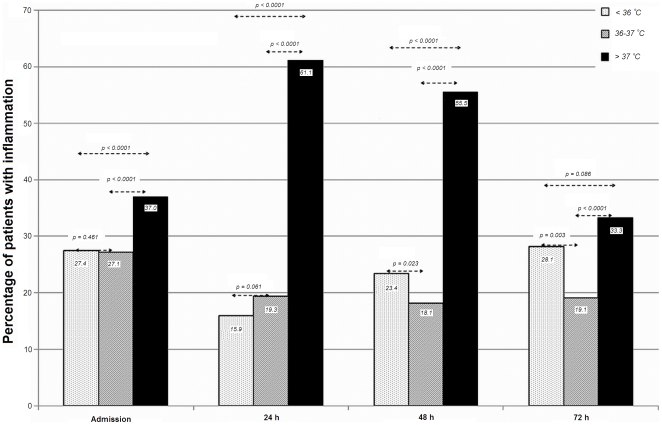
Patients with high temperatures (>37°C) have more inflammation in all time periods analyzed.

**Figure 5:**
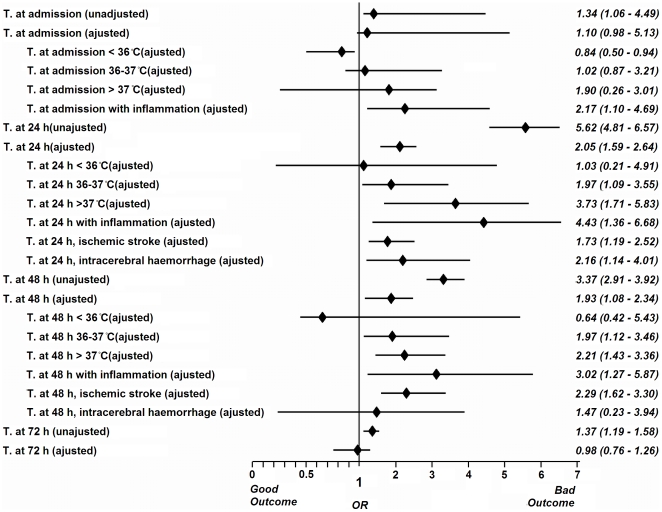
Odds ratio and confidence intervals 95% of all the groups analyzed.

Among patients with basal high temperature, intercurrent infection was detected in 0.7% of them on admission, 6.2% at 24 hours, 18.0% at 48 hours and 21.7% at 72 hours. The presence of systemic infection was higher in the group of patients with high temperature (>37 C) compared with any other groups 36–37 C and <36 (p<0,0001) (**Figure 4**). In the subgroup of patients with systemic inflammation, high temperature was associated with poor outcome on admission (OR 2.17, 95% CI 1.10–4.69, p = 0.009), at 24 hours (OR 4.43, 95% CI 1.36–6.68, p<0.0001) and at 48 hours (OR 3.02, 95% CI 1.17–5.87, p = 0.001). **Figure 5** shows the result of all multivariate models analyzed.

## Discussion

### The most novel observations demonstrated in our study are the following

#### 1. Acute cerebrovascular disease is associated with increased body temperature

Temperature rise is probably due to brain injury and not to systemic complications, since it was analyzed at 8 hours on average from stroke onset (including 16.4% of patients who had a wake-up stroke). Differences in temperature among patients and controls exist despite the fact that 16.3% of the patients were previously taking antiplatelet therapy, mostly aspirin. One limitation to this conclusion is that we used subjects with a different risk factor profile as the control group, but the differences were also significant in 2571 patients at 3 months (36.21±0.54°C, p<0.0001) compared to the temperature on admission (data not shown previously), although at 3 months we lost most severe patients for the analysis and 62.8% of patients were taking antiplatelet agents.

Our data are consistent with experimental studies that show an increase of pro-inflammatory cytokines and leukocytes surrounding the infarcted tissue [15], which may be responsible for hypothalamic neuronal stimulation. Regional temperature increase surrounding the infarcted area was also found using MRI spectroscopy [16], although its relationship with outcome has not been confirmed.

Contrary to the opinion that early hyperthermia, probably of neurogenic origin rather than systemic, is associated with large lesions [17], no association between temperature on admission and stroke severity (**Figure 2**), or volume of lesion was found (data not shown). Therefore, this effect may be due to metabolic (potentially modifiable) rather than structural mechanisms.

#### 2. The therapeutic window for neuroprotection mediated by hypothermia is shorter than 24 hours

In our study the decrease in NIHSS score observed in the group of patients with low temperature on admission is only evident in the first 24 hours, and, in multivariate analysis, only temperature below 36°C was independently associated with good outcome at 3 months.

In animal models, the ability of hypothermia to confer neuroprotection was found to be directly dependent on the delay of treatment initiation [18], and in clinical practice, prolonged hypothermia is used because in most patients the decrease of temperature is induced several hours after the stroke onset [19]. Two clinical trials in patients with cardiac arrest showed that when hypothermia was started within 2 hours after the event, a significant reduction in neurological deficit was obtained [20], [21]. Our observation is also supported by experimental data. Many of the mechanisms of neuroprotection associated with hypothermia, particularly neurotoxicity and inflammation, appear minutes after induction of ischemia and have a short duration [22].

In regard to optimal duration of hypothermia induction, preclinical data have demonstrated that hypothermia for 48 hours initiated 1 hour after permanent MCA occlusion in rats provided better functional and morphologic outcomes than hypothermia for 24 or 12 hours, [23], therefore it would seem reasonable to think that longer hypothermia time could induce better results, however, to date there is no clear clinical evidence to demonstrate such effects. Our data, in agreement with another recent clinical study [11], show that the window for neuroprotection mediated hypothermia is less than 24 hours. Our observations may be also interesting for future clinical trials, since maintaining hypothermia for several days increases patient discomfort and complications derived from the process [24]. Under our opinion multiple factors can determine the optimal duration of hypothermia in patients, such as the severity of ischemia, occurrence of reperfusion, time of the hypothermia induction after ischemia, risk of complication because of hypothermia, etc [3]. One small phase II trial is currently comparing the feasibility of cooling for 12 and 24 hours in patients with acute ischemic stroke (http://www.trialregister.nl/trialreg/admin/rctview.asp?TC=2616.

Another important consideration is that temperature on admission that was associated with lower brain damage in our study was between 34.6 and 36°C. This observation is in line with those studies that show that temperatures between 35 and 35.5°C are tolerable and safe in clinical practice, however there is clear no evidence of greater efficacy with temperatures below 35°C [6], [25]. Some clinical studies have demonstrated the possibility to achieve hypothermia between 33°C and 35°C [26], [27], [28] and control a long lasting warming up [29] with the aim of increase the neuroprotective efficacy of hypothermia. Unfortunately, discomfort and shivering increase with lower temperatures, and cooling to these levels generally requires sedation, mechanical ventilation, and admission to an intensive care unit (ICU) [30] with the risk of eliminating the benefits of the hypothermia treatment. Nowadays, a new clinical trial in progress (http://public.ukcrn.org.uk/search/StudyDetail.aspx?StudyID=7853) is being conducted to find out if cooling patients to either 33°C or 35°C, from a normal body temperature of around 37°C could become a new treatment for reducing death and disability following acute ischemic stroke.

#### 3. The window to prevent brain damage mediated by hyperthermia persists for at least 48 hours and is associated with inflammatory mechanisms

This study confirms previous findings [4], [5], [6], [7], [8], [9], [10], [11], [12] regarding the association between hyperthermia and poor outcome in acute cerebrovascular disease. However, this association is demonstrated only after admission and during the first 48 hours. The discrepancy between these data and those studies which find association with temperature on admission [5], [7], [10] is probably due to the shortening of latency between stroke onset and arrival at hospital. In our study 32.9% of patients arrived within the first 3 hours and 53.8% within the first 6 hours after stroke onset, and there was no relationship between NIHSS and temperature in patients admitted within 3 hours (Spearman coefficient = −0.007), whereas a significant association exists for patients admitted to hospital after 9 hours (Spearman coefficient = 0.169, data not shown). The PAIS study [12] analyzed patients admitted before 12 hours from stroke onset. In this study, the temperature on admission was not associated with outcome, but temperature rise in the following hours after admission was associated with poor outcome, as it also happens in the VISTA study [11].

The presence of systemic inflammation significantly increases the association between high temperatures and poor outcome, and this association is also demonstrated with basal temperatures. This aspect, which must be confirmed, may have clinical relevance for selecting patients with high basal temperatures and inflammatory response as candidates for more intensive antipyretic treatments or even early hypothermic therapy. Systemic infection is associated with high temperature only after 24 hours, but not during the first hours after stroke onset.

The robust association of inflammation with hyperthermia and poor outcome observed in our analysis explain why the blockade of the inflammatory response is considered one of the most important mechanisms associated with the beneficial effects of hypothermia in ischemic stroke [31]. In addition, these data confirm that blood levels of cytokines like TNF-α and IL-6 act as good biomarkers of final infarct volume and early neurological deterioration [32]

It is well established that after glia activation, increase of pro-inflammatory cytokines and neutrophils appear within hours of focal cerebral ischemia, peaking 1–2 days later in the rodent, and are then replaced by monocytes/macrophages at 3–7 days. Leukocytes enter injured tissue and contribute to secondary injury by releasing reactive oxygen species (ROS), generating lipid mediators, activating thrombosis, damaging the blood brain barrier (BBB), increasing cerebral edema, and plugging the cerebral microvasculature [15], [33]. However this long temporal profile does not explain why infection is associated with high temperature mainly 24–48 h after stroke and why the therapeutic window for neuroprotection mediated by hypothermia is less than 24 hours. It may, in part, be determined by the dual role of inflammation on ischemic tissue. Besides the deleterious effect of inflammation response, the phagocytic activity of activated microglia and macrophages exerts beneficial effects in the long term and may be necessary for tissue healing [31]. Therefore, delayed hypothermia may adversely impact physiological conditions on those processes necessary for tissue recovery such as inflammation response.

#### 4. Body temperature in relation to stroke outcome acts as a continuous variable, uncategorized, both in ischemia and ICH

**Figure 6:**
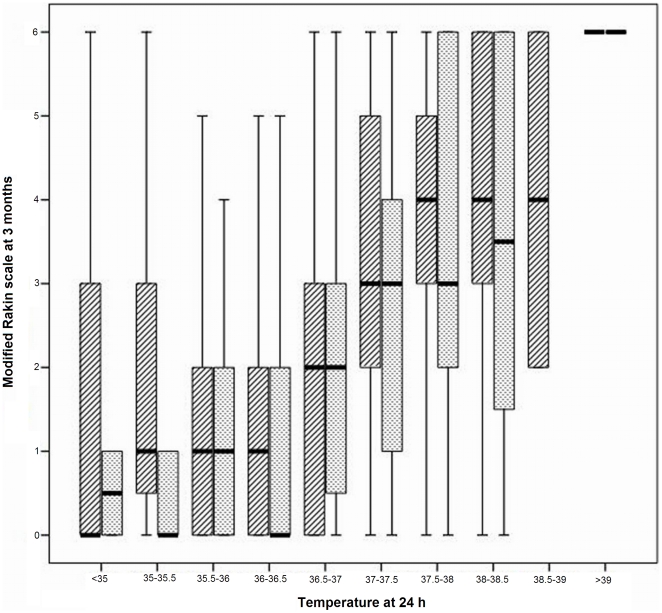
Modified Rankin scale at 3 months in relation to temperature intervals of 0.5°C from 34.5 to 39.5°C. The relationship is similar for patients with ischemic stroke (striped columns) and intracerebral hemorrhage (dotted columns).

Most of the published studies tend to categorize temperature, considering hyperthermia from 37 to 38°C [4], [5], [6], [7], [8], [9], [10], [11], [12], limiting the benefit of treatment for patients with temperatures higher than the temperature threshold. Our study demonstrates that temperature is a continuous variable (see **Figure 2**) and its relationship with outcome at 3 months is linear from 36.5°C, both in IS and in ICH (**Figure 6**).

Although the strength of the association between temperature and outcome is much higher for patients with IS, some studies pointed out its influence on ICH. The temperature rise during the first 24 hours is higher in ICH [12], and in animal models of brain hemorrhage, hypothermia demonstrated benefit in relation to brain edema reduction, but not to hematoma volume [34].

If our results are confirmed, an indication for antipyretic treatment should start as soon as possible, both for IS and ICS, starting with temperatures ≥36.5°C, with 36°C or below as a therapeutic target.

#### 5. Basal body temperature does not predict the efficacy of thrombolytic therapy

In 121 patients treated with tPA according to SITS-MOST criteria, the same benefit was obtained in patients with low, normal or high temperature at admission (good outcome at 3 months: 65, 44 and 63%, p = 0.087). However, the persistence of high temperatures at 24 and 48 hours conditioned a poor outcome at 3 months (84 and 75% respectively).

In vitro studies showed an increase in fibrinolytic activity at high temperatures [35], and in a recent study in 111 patients treated with tPA, high temperatures at admission were associated with a better outcome [36]. These results have not been validated by other studies [12], [36], [37], but have shown results similar to ours [38].

As a result, basal body temperature should not condition the administration of thrombolytic treatment, but in these patients, the temperature control must be strict and temperature rise during the first 48 hours should be avoided. In fact, recently we have demonstrated that high body temperature (≥37°C) is associated with lack of recanalization, greater hypodensity volume and worse outcome in stroke patients treated with tPA, and risk of hemorrhagic transformation in patients untreated with tPA, at 24 hours but not at admission [38], [39].

Our study has some strong points from the analysis of a large group of non-selected patients treated according to international guidelines protocols in a stroke unit with certificated staff. Although this is the analysis of a database, the selection of the variables and the statistical methods were designed and approved prior to analysis of the results. No data in this study was obtained secondarily to the findings. The main limitation of our study comes from the method for determining the temperature, the axillary temperature measurement requires careful attention by the medical staff and this procedure is not always followed.

In summary, the inherent limitations of recanalization procedures, the failure of other neuroprotective therapies, and the possibility to control temperature and other physiological variables in all patients, make the control of temperature one of the most successful treatments in patients with an acute cerebrovascular event. The results of our study suggest that the influence of temperature on brain damage is time-dependent. During the first hours, hyperthermia is infrequent and is associated with intracerebral –non-systemic- mechanisms, so its early control may block the ischemic cascade and lead to neuroprotective effects. Lowering the temperature below 36°C appears to have significant benefits. In contrast, later high temperatures are associated with inflammatory mechanisms and seem to have a systemic origin. Its control in a wider window of 48–72 hours may prevent the progression of brain damage.
